# Recent Advances in Medium-Chain Triglycerides in Chronic Disease Prevention

**DOI:** 10.3390/nu18132133

**Published:** 2026-07-01

**Authors:** Yonghui Yu, Wanxin Ya, Jingjie Zhang, Jing Wang, Baoguo Sun

**Affiliations:** 1 Key Laboratory of Geriatric Nutrition and Health, Ministry of Education, Beijing Technology and Business University, Beijing 100048, China; yonghuiwh@126.com (Y.Y.);; 2 China-Canada Joint Lab of Food Nutrition and Health, Beijing Technology and Business University, Beijing 100048, China; 19544556517@163.com

**Keywords:** medium-chain triglycerides, metabolic diseases, neurodegenerative diseases, gut microbiota, muscle function, precision nutrition

## Abstract

Medium-chain triglycerides (MCTs) are functional lipids with unique physicochemical properties and metabolic advantages. Recently, their regulatory roles in various chronic diseases have attracted considerable attention. This review systematically summarizes recent research progress and the proposed mechanisms of MCTs and their metabolites in metabolic diseases, neurological disorders, gut health, and muscle function. In the metabolic field, MCTs offer potential nutritional strategies for managing obesity, type 2 diabetes mellitus (T2DM), and various metabolic liver diseases. These effects are primarily mediated by improving insulin sensitivity, regulating lipid metabolism, and modulating energy expenditure. In neurological diseases, MCTs demonstrate potential for preventing and treating Alzheimer’s disease (AD), Parkinson’s disease (PD), and epilepsy through multiple pathways, including ketogenic energy supply, anti-inflammatory and antioxidant effects, and mitochondrial protection. Regarding gut health, MCTs and their derivatives may benefit digestive health by modulating gut microbiota and enhancing barrier function. For muscle health, MCTs help optimize energy metabolism and protein homeostasis, showing promise for countering sarcopenia and improving exercise performance. In conclusion, the prospects for MCTs are broad. Future research should focus on promoting their scientific application in precision nutrition and disease therapy, and more rigorous clinical trials are needed to confirm their efficacy and safety.

## 1. Introduction

As the global burden of chronic diseases, including obesity, diabetes, and neurodegenerative disorders, continues to rise, nutritional interventions have emerged as promising complementary strategies for prevention and management. Among these, medium-chain triglycerides (MCTs) have gained increasing attention due to their unique metabolic properties. Unlike long-chain triglycerides (LCTs), which dominate Western diets and are readily stored in adipose tissue, MCTs are rapidly absorbed, transported directly to the liver via the portal vein, and preferentially oxidized for energy. This difference positions MCTs as a potential dietary strategy to counteract key drivers of chronic diseases, such as insulin resistance, hepatic steatosis, neurocognitive decline, and gut barrier dysfunction. Furthermore, MCTs efficiently produce ketone bodies, which not only serve as an alternative fuel for the brain but also act as signaling molecules that modulate inflammation, oxidative stress, and mitochondrial function. These mechanisms provide a solid scientific basis for exploring MCTs as a multifunctional nutritional intervention. Previous reviews have largely focused on isolated aspects of MCT biology or solely on ketogenic mechanisms without considering their broader pleiotropic effects, and systematic reviews are scarce and outdated. This review provides an analysis of selected relevant mechanisms and clinical applications of MCTs over recent years across four major health domains, aiming to inform the rational use of MCTs in precision nutrition and clinical practice.

MCTs are structurally modified lipids formed by esterification of three molecules of medium-chain fatty acids (MCFAs) with one molecule of glycerol [1]. These triglycerides are characterized by rapid absorption and easy β-oxidation, making them ideal carriers for functional dietary supplements [2]. The definition of MCFAs based on carbon chain length remains debated; traditionally, they are defined as containing 6–12 carbon atoms, but industrial standards are evolving dynamically with production technologies. Natural sources are mainly palm kernel oil and coconut oil, in which MCFAs account for more than 50% of total fatty acids, while milk fat contains about 4–12% MCFAs [3]. In biosynthetic pathways, affected by the acetyl-CoA binding mechanism, natural MCFAs are limited to a few chain lengths. The primary MCFAs are hexanoic acid (C6:0), octanoic acid (C8:0), decanoic acid (C10:0), and lauric acid (C12:0) [4]. From the point of view of biochemistry and structural representation, C8 and C10 fatty acids are recognized as the most representative MCFA components [5], constituting 40–50% and 25–35%, respectively. These two fractions together account for 65–85% of MCFA composition [6]. Their favorable physicochemical properties facilitate commercial development and widespread industrial production.

MCTs are colorless, odorless and tasteless liquids at room temperature and can efficiently dissolve fat-soluble bioactive components (e.g., vitamins, antioxidants) [7]. MCTs have low melting and boiling points [8], and are characterized by high efficiency and low toxicity [9]. Upon complete oxidation, their energy density is approximately 9 kcal/g upon complete oxidation [10], which is 2.25 times that of carbohydrates or proteins [11]. Compared to animal or plant-derived fats, MCTs have lower smoke points (140–160 °C) [8]. When heated above the smoke point, MCTs generate harmful volatile organic compounds, including acrolein and malondialdehyde, with volatile emissions reaching up to 2.3 times those of LCTs [12]. These limitations can be improved by chemical structural modifications, such as developing medium- and long-chain triglycerides (MLCTs) synthesized from MCFAs and LCFAs [13]. Due to their short carbon chains and relatively small molecular size, MCTs are rapidly metabolized, transported and absorbed in the human body, serving mainly as an instant energy source [14]. Their metabolic and energy supply rates are inversely related to the length of the carbon chain: longer chains slow down metabolism and delay energy delivery [4]. C6 has the shortest carbon chain and the fastest metabolic turnover, but it often causes gastrointestinal discomfort, such as nausea, vomiting, and abdominal distension [15]. Additionally, the taste is poor. C12 has a longer chain and slower metabolic clearance, although it is physiologically better tolerated. Therefore, daily intake of MCTs containing C8 and C10 is generally recommended. One clinical study has confirmed that 30 g of MCT sodium daily for 30 consecutive days in healthy individuals had no adverse effects on blood glucose, insulin, triglyceride cholesterol, free fatty acids, body weight or body mass index (BMI) (Table 1) [16].

## 2. Metabolism and Characteristics of MCTs

MCTs have unique absorption and transport patterns compared to LCTs, which may increase satiety, reduce caloric intake [46], and improve lipid metabolism [47]. Digestion of LCTs follows a conventional lipid metabolism pathway: in the small intestine, enzymatic hydrolysis generates long-chain fatty acids (LCFAs) and 2-monoglycerides. Free LCFAs passively diffuse into intestinal epithelial cells, where they are re-esterified into triglycerides. These triglycerides bind to cholesterol and lipoproteins to form chylomicrons, which enter the lymphatic system and are transported to the liver via the hepatic artery. In the liver, carnitine acyltransferase is involved in LCT metabolism. The re-synthesized triglycerides are stored mainly in adipose tissue or liver cells. Because the liver has a limited capacity to store triglycerides, excess triglycerides are exported to peripheral tissue as very-low-density lipoproteins (VLDLs) [48,49]. In contrast, the absorption mechanisms of MCTs are more similar to those of glucose than to lipids. The MCFAs produced by hydrolysis are highly oxidizable and do not rely on the bile salt pathway. Their absorption requires only small amounts of pancreatic enzymes [37]. Absorbed MCFAs do not need to be re-esterified or assembled into chylomicrons. Instead, they are transported directly via the portal vein to the liver. In the liver cells, MCFAs enter mitochondria without carnitine mediation and undergo rapid β-oxidation to acetyl-CoA [50]. Excess acetyl-CoA is readily converted to ketone bodies [51]. Acetone is excreted via respiration or urine, whereas β-hydroxybutyrate (βHB) and acetoacetate (AcAc) enter the circulation via monocarboxylic acid transporters for use by peripheral tissues [52].

The metabolic advantages of MCTs are reflected in their ability to provide a fast and efficient energy substrate. Their hydrolysis in the intestinal cavity is faster and more complete than LCTs. Bile salt is not necessary for optimal absorption, allowing complete hydrolysis and absorption in the small intestine while bypassing chylomicron assembly and the lymphatic transport pathway. These properties ensure adequate oxidative utilization while reducing adipose tissue deposition mediated by hormone-sensitive lipase (HSL). Consequently, MCTs serve as a prioritized rapid energy source for brain, muscle, liver, and heart tissue [4,14,53,54,55,56]. When ingested together with LCTs, MCTs exhibit better intestinal absorption dynamics while accelerating the hydrolysis of LCTs [4]. This synergistic effect promotes β-oxidation of LCFAs, thereby improving lipid metabolism [57]. Notably, in the absence of LCFAs, C12 can be metabolized via LCFA-specific pathways [58].

## 3. Recent Advances in the Role of MCTs in Metabolic Diseases

Metabolic syndrome is a complex clinical condition characterized by the clustering of multiple cardiovascular risk factors. Its core diagnostic criteria include abdominal obesity, high blood pressure, hyperglycemia, and dyslipidemia (mainly elevated triglycerides and reduced high-density lipoprotein cholesterol, HDL-C) [59]. The pathophysiological basis of this syndrome and its related diseases, such as obesity, type 2 diabetes mellitus (T2DM), and various metabolic liver diseases, involves multiple interacting mechanisms, with insulin resistance considered a key driver [60]. Insulin resistance interacts with chronic low-grade inflammation and oxidative stress, forming a vicious cycle [61,62]. MCTs have shown promise in the management of metabolic diseases as a potential nutritional intervention strategy. They regulate glucose and fat metabolism in multiple ways, including mitigating obesity, improving insulin sensitivity, and exerting hepatoprotective effects.

### 3.1. Alleviating Obesity

**Figure 1:**
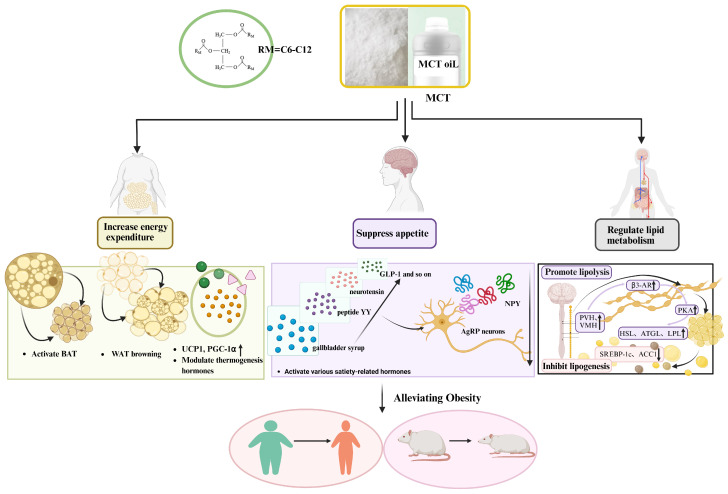
Diagram illustrating the three major mechanisms through which MCTs exert weight-reducing effects. The main mechanisms of MCTs reducing weight: Firstly, increasing energy expenditure: MCTs induce thermogenic markers in brown adipose tissue (BAT) and subcutaneous white adipose tissue (WAT) by upregulating uncoupling protein 1 (UCP1) and peroxisome proliferator-activated receptor gamma coactivator 1-alpha (PGC-1α), as well as modulating thermogenic hormones, thereby enhancing energy consumption. Secondly, suppressing appetite: MCTs reduce food intake by stimulating the release of satiety hormones (e.g., peptide YY, glucagon-like peptide-1 (GLP-1)) and inhibiting expression of orexigenic neuropeptides (e.g., neuropeptide Y (NPY), agouti-related protein (AgRP)) in the hypothalamus. Thirdly, regulating lipid metabolism: MCTs activate the sympathetic nervous system, enhance β3-AR (beta-3 adrenergic receptor) and PKA (protein kinase A) signaling, and increase lipase activity (HSL, adipose triglyceride lipase (ATGL), lipoprotein lipase (LPL)) to promote fat breakdown; concurrently, MCTs inhibit lipogenesis by downregulating key lipogenic proteins (e.g., sterol regulatory element-binding protein 1c (SREBP-1c), acetyl-CoA carboxylase (ACC)). The figure was created with BioRender.com.

Obesity has become an important risk factor for many chronic metabolic diseases. However, currently approved anti-obesity drugs often have significant side effects or limited efficacy. In contrast, nutritional interventions—particularly supplementation—are increasingly becoming a high-profile strategy in weight management because of their safety and sustainability. Recent research indicates that MCTs exert weight-reducing effects through three primary, synchronized mechanisms: increasing energy expenditure, suppressing appetite, and regulating fat metabolism (Figure 1).

#### 3.1.1. Increasing Energy Expenditure (EE)

MCTs reduce body weight by stimulating thermogenesis and increasing energy expenditure. Animal studies have shown that MCTs stimulate brown adipose tissue (BAT) thermogenesis and induce thermogenic markers in subcutaneous white adipose tissue (WAT) [47,63,64]. The underlying mechanism may promote WAT thermogenesis and increase energy consumption by activating the adenosine monophosphate-activated protein kinase (AMPK) pathway and upregulating the expression of core thermogenic factors like uncoupling protein 1 (UCP1) and peroxisome proliferator-activated receptor gamma coactivator 1-alpha (PGC-1α) (Table 2) [65]. As shown in Table 1, a two-week clinical intervention showed that MCT intake significantly increased postprandial EE and promoted the oxidation of dietary LCTs in sedentary obese individuals [17]. Another four-week clinical intervention found that the effect of MCTs on EE in obese patients varied by sex and baseline body weight. Among males, lighter-weight individuals showed greater EE and fat oxidation [18]; in women, EE and fat oxidation were more pronounced in those with higher body weight [19].

#### 3.1.2. Appetite Suppression

MCTs are also able to suppress appetite through various neuroendocrine mechanisms, thereby reducing food intake and achieving weight control. MCTs activate multiple satiety-related hormones, including cholecystokinin, leptin, peptide YY, gastric inhibitory peptide, neurotensin, pancreatic polypeptide, and glucagon-like peptide-1 (GLP-1) [86,87,88]. They reduce energy intake by delaying gastric emptying or increasing the concentration of β-hydroxybutyrate (Table 2) [66]. The primary mechanism involves β-hydroxybutyrate acting as a signaling molecule that suppresses the expression of appetite-stimulating neuropeptides such as neuropeptide Y (NPY) and agouti-related protein (AgRP) in the hypothalamus, while activating the glutathione signaling pathway associated with satiety [89,90].

#### 3.1.3. Regulating Fat Metabolism

MCTs can also control body weight by promoting fat breakdown and inhibiting fat production, thereby reducing lipid accumulation. In promoting fat breakdown, MCTs may enhance sympathetic nervous system activity and increase neural innervation density in the hypothalamic paraventricular nucleus (PVH) and the ventromedial nucleus (VMH) [56]. This in turn induces the release of norepinephrine and activates the β3-adrenergic receptor (β3-AR) and its downstream protein kinase A (PKA) signaling pathway [63]. Subsequently, it enhances the phosphorylation of Akt protein in muscle and adipose tissue, thereby increasing the activity of HSL, adipose triglyceride lipase (ATGL), and lipoprotein lipase (LPL) to promote adipocyte lipolysis (Table 2) [67]. Regarding the inhibition of lipogenesis, MCTs downregulate the expression of key lipogenic proteins, including sterol regulatory element-binding protein-1c (SREBP-1c), acetyl-CoA carboxylase 1 (ACC1), fatty acid synthase, and stearoyl-CoA desaturase 1 (SCD1) [68]. This inhibits de novo fat synthesis.

### 3.2. Enhancing Insulin Sensitivity and Glucose Homeostasis

Insulin resistance is a central part of the development and progression of T2DM. It stems primarily from abnormal lipid metabolism, in which the accumulation of triglycerides and ceramides interferes with insulin signaling [91]. Adipose tissue inflammation and cellular stress further exacerbate insulin dysfunction. Multiple mechanisms interact to form a complex regulatory network that affects metabolic status in various organs [92]. Studies have shown that MCFAs, especially the C8 and C10 components, may improve insulin sensitivity and blood glucose homeostasis by regulating key cell signaling pathways and metabolism.

At the mechanistic level, MCFAs of different chain lengths may function through distinct signaling pathways. C10 enhances the transduction efficiency of the core insulin signaling pathway, phosphatidylinositol 3-kinase/protein kinase B (PI3K/Akt). It does so by activating peroxisome proliferator-activated receptor γ (PPARγ) and inhibiting phosphorylation at the PPARγ Ser237 site. C10 also activates PPARδ, upregulates the expression of UCP3 in skeletal muscle, and promotes fatty acid oxidation without affecting insulin signal (Table 2) [69,70]. Notably, both C8 and C10 restore the expression of metabolic genes in adipocytes damaged by inflammation by increasing histone acetylation, with C10 showing a more significant effect [93]. Compared with C8 and C10, C12 exhibits tissue-specific action. In the liver, C12 may induce hepatic insulin resistance by upregulating hepatocyte nuclear factor 4α (HNF4α)-dependent selenoprotein P expression, thereby interfering with Akt phosphorylation [94]. In skeletal muscle, C12 promotes mitochondrial biogenesis by activating the peroxisome PGC-1α/PPARγ pathway and upregulates the expression of glucose transporters (GLUT-1 and GLUT-3), ultimately enhancing glucose uptake capacity [95].

Mechanistic studies on how MCTs regulate related signaling pathways lay the theoretical foundation for their clinical application. A study involving patients with non-insulin-dependent diabetes mellitus found that while MCTs had limited effects on improving fasting blood glucose or long-term glycemic markers, they effectively attenuated postprandial glucose fluctuations, suggesting utility in preventing postprandial metabolic stress (Table 1) [20]. In obese individuals, an MCT-based diet was more effective in improving the homeostasis model assessment of insulin resistance (HOMA-IR) index, oral glucose tolerance test (OGTT) metrics, insulin sensitivity [69,70], and HDL-C levels [96]. Furthermore, studies indicate that even replacing a small amount of dietary LCTs with MCTs can effectively buffer against high-fat-diet-induced insulin resistance and help maintain glucose metabolic balance [97].

### 3.3. Ameliorating Metabolism-Related Liver Diseases

The liver is the core organ for energy and substance metabolism. MCTs can bypass certain impaired pathways of LCT metabolism, providing rapid and efficient energy substrates, balancing lipid metabolism, and reducing fat accumulation. They also exert hepatoprotective effects through multiple mechanisms, demonstrating unique value in the management of metabolic liver diseases.

For specific inherited metabolic liver diseases, MCTs can serve as important alternative energy substrates. As summarized in Table 1, diseases involving metabolic defects in LCFAs, such as carnitine palmitoyltransferase 1A (CPT1A) deficiency, MCTs can directly provide substrates for rapid mitochondrial β-oxidation, thereby bypassing the defective metabolic process (Table 2) [21]. For neonatal intrahepatic cholestasis caused by citrin deficiency, multiple clinical reports confirm that MCT intervention helps improve bile accumulation and clotting function, and restore liver energy and redox balance [22]. In children with biliary atresia, MCTs can significantly increase energy intake, supporting preoperative nutrition and growth [23,24].

For acquired metabolic liver disease, MCTs demonstrate potential for multi-target intervention in metabolic dysfunction-associated steatotic liver disease (MASLD) and its progressive form, metabolic dysfunction-associated steatohepatitis (MASH). The core mechanism involves reshaping liver lipid metabolism homeostasis. On one hand, MCTs can activate signaling pathways such as the farnesoid X receptor (FXR) and peroxisome proliferator-activated receptor α (PPARα) [98]. They upregulate levels of hyodeoxycholic acid (HDCA) and inhibit the expression of triglycerides (TGs) [71], thereby promoting fatty acid β-oxidation and carrier protein (e.g., Apoa4/Apoc2)-mediated liver lipid clearance [99,100]. On the other hand, MCTs significantly inhibit the activity of sterol regulatory element-binding protein-1c (SREBP-1c) and diacylglycerol acyltransferase 1 (DGAT1), suppressing lipogenesis [99,100,101]. The combined action of these pathways systematically reverses hepatic lipid accumulation.

Beyond metabolic regulation, MCTs also protect the liver through anti-inflammatory, antioxidant, and cytoprotective mechanisms. Studies indicate that MCTs can ameliorate the local hepatic inflammatory microenvironment and reduce inflammatory responses and hepatocyte death induced by various injurious factors. This effect is achieved by inhibiting key pathways in necroptosis (receptor-interaction protein kinase 1/3 and mixed lineage kinase domain-like protein, RIP1/RIP3/MLKL), as well as suppressing macrophage overactivation induced by lipotoxic stress. MCTs can also induce the expression of anti-stress proteins such as heat shock protein 70 and activate receptors like GPR84 [102,103]. Furthermore, MCT intervention can effectively reduce the level of lipotoxic signaling molecules such as ceramides and diacylglycerol in the liver, blocking abnormal membrane transitions of protein kinase C-ε (PKC-ε) and playing a hepatoprotective role. For some indicators, the protective effect of MCTs is even comparable to that of classical drugs (Table 2) [72]. These findings reveal multiple pathways through which MCTs directly maintain hepatocyte homeostasis at the molecular level.

The mechanisms underlying MCTs’ effects on obesity, insulin sensitivity, and liver diseases are supported by a combination of preclinical studies (cell and animal models) and small- to medium-sized clinical trials. Most clinical studies have limited sample sizes and short intervention periods. Large-scale, long-term randomized controlled trials are lacking. The differential effects of C8, C10, and C12 on insulin signaling (particularly the potential adverse effect of C12 in the liver) warrant further investigation. For inherited metabolic liver diseases, evidence is derived from case reports and small studies, representing a lower level of evidence but with clear therapeutic rationale.

## 4. Recent Advances in MCTs in Neurological Diseases

MCTs exert neuroprotective effects primarily through their potent ketogenic properties. Its metabolite, MCFAs, can be rapidly oxidized to acetyl-CoA in liver mitochondria independently of insulin, thereby promoting the synthesis of ketone bodies (β-HB and AcAc) [104]. These ketones cross the blood–brain barrier (BBB) and act as alternative energy substrates in the brain, where they are reconverted to acetyl-CoA and enter the tricarboxylic acid (TCA) cycle. This direct neuronal energy supply mechanism is especially important under conditions of impaired brain glucose metabolism [6]. A review of 17 studies on ketogenic diets for neurodegenerative disorders reported benefits including improved cognitive outcomes, enhanced brain ketone utilization, modulated cerebral blood flow, reduced inflammation, and improved metabolic parameters [105]. Beyond their ketogenic effect, MCTs confer therapeutic value through multiple additional mechanisms. These primarily include anti-inflammatory actions (e.g., via NF-κB/p38-MAPK signaling) [106], protection of mitochondrial function (e.g., via upregulation of the PGC1α-SIRT3-UCP2 axis and modulation of PPARγ/RXR signaling) [107,108,109], mitigation of oxidative stress (e.g., via glutathione, GSH) [110], modulation of mTORC1 signaling [111], and elevation of inhibitory neurotransmitter levels (e.g., GABA) [112].

### 4.1. Mild Cognitive Impairment (MCI) and AD

**Figure 2:**
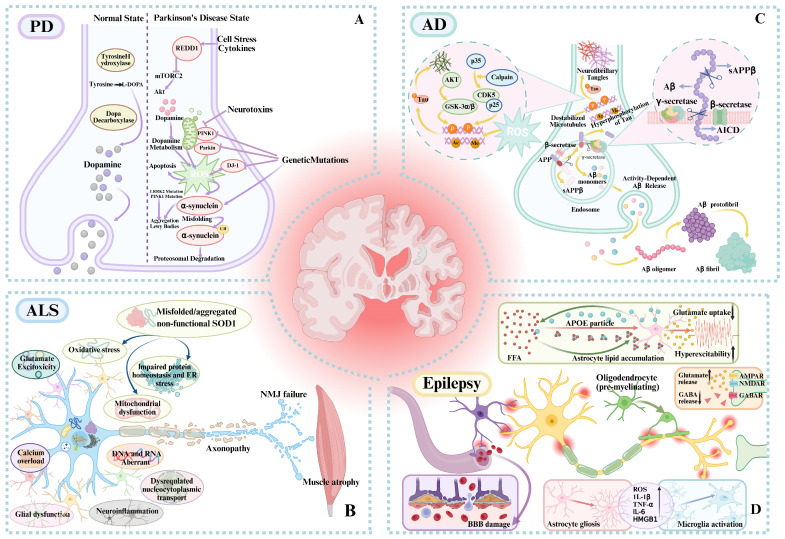
Schematic diagram of the core pathological mechanisms of four neurological diseases and potential intervention sites of MCTs. It summarizes the core pathogenic mechanisms of Parkinson’s disease (PD), amyotrophic lateral sclerosis (ALS), Alzheimer’s disease (AD), and epilepsy. These diseases involve key pathological pathways such as neuroinflammation, oxidative stress, mitochondrial dysfunction, and impaired proteostasis. (**A**) PD: Characterized by loss of dopaminergic neurons in the substantia nigra, involving dopamine metabolism dysfunction, α-synuclein pathology, impaired mitophagy (PINK1/Parkin), neuroinflammation, and proteasomal dysfunction. (**B**) ALS: Manifests as motor neuron degeneration. Mechanisms include protein misfolding/aggregation (e.g., superoxide dismutase 1 (SOD1)), glutamate excitotoxicity, impaired axonal transport, and glia-mediated neuroinflammation. (**C**) AD: Core pathologies are amyloid beta (Aβ) plaques and neurofibrillary tangles. The figure illustrates the Aβ generation cascade from amyloid precursor protein (APP) cleavage to oligomerization and fibrillization, and the Tau protein hyperphosphorylation process mediated by kinases such as glycogen synthase kinase 3 beta (GSK-3β) and cyclin-dependent kinase 5 (CDK5). (**D**) Epilepsy: Focuses on neuronal network hyperexcitability. Mechanisms involve glutamate/gamma-aminobutyric acid (GABA) imbalance, astrocyte dysfunction (e.g., impaired glutamate uptake), blood–brain barrier damage, and associated neuroinflammation (e.g., release of interleukin 1 beta (IL-1β), high mobility group box 1 (HMGB1)). MCTs may exert potential intervention effects at multiple sites shown in the figure by providing efficient ketone bodies as an energy source, improving mitochondrial function, reducing oxidative stress and neuroinflammation, and potentially modulating excitatory/inhibitory neurotransmitter balance. The figure was created with BioRender.com.

MCI represents a transitional state between normal age-related cognitive changes and early-stage dementia. It is characterized by memory decline, reduced attention and concentration, language difficulties, and impaired executive function [113]. MCI patients carrying genetic risk factors such as the apolipoprotein E4 (APOE ε4) allele, which may progress to AD. AD manifests as cognitive decline, memory impairment, disorientation, and behavioral changes, with core pathologies including the deposition of β-amyloid (Aβ) plaques and the abnormal aggregation of tau protein [114] (Figure 2B). These protein lesions directly damage neurons, triggering severe oxidative stress and neuroinflammation, leading to widespread cell damage [115]. Glutamate signaling supported by astrocytes in key brain regions such as the hippocampus also becomes disordered. This further exacerbates neuronal dysfunction and synapse loss, thereby worsening cognitive decline [116].

Clinical studies confirm that MCT supplementation enhances brain energy metabolism and improves cognitive function in MCI and AD patients. As shown in Table 1, daily supplementation of 30 g of ketogenic MCTs for 6 months significantly increased blood ketone body and MCFA levels in MCI patients, with a favorable safety profile [25]. Another study using a higher dose of 42 g/day MCT oil for 6 months reported stable or improved cognitive function in 80% of MCI patients, and the effect was further enhanced after 9 months [26]. Doses of 20–70 g/day have also been shown to improve cognitive function in patients with mild to moderate AD and MCI, primarily by enhancing the brain’s utilization of ketones. No significant adverse effects were observed in these trials [27]. A meta-analysis of 21 studies concluded that MCT intervention consistently raises plasma ketone levels and cerebral metabolic rate, although cognitive improvements were confined to specific domains [73].

From a mechanistic perspective, MCTs target AD pathology through multiple pathways. They directly enhance the activity of insulin-degrading enzyme (IDE) and activate GPR84 and other pathways to promote Aβ clearance, exerting neuroprotective effects (Table 2) [74]. Although supplementation with MCTs increased plasma ketone body levels in both APOE ε4 carriers and non-carriers, the response was better in non-carriers [117]; carriers showed limited improvement in core domains such as memory [118]. Synergistic effects have been observed: co-administration of MCFAs with docosahexaenoic acid (DHA) enhances hepatic Aβ clearance, significantly reducing Aβ burden in the brain and serum [98].

Distinct MCFAs exert their effects through different mechanisms. C8 can effectively stimulate the liver and astrocytes to produce ketones, thereby improving whole-body ketone body levels and enhancing brain energy metabolism. C10 supports neuronal function by promoting glycolysis and lactic acid production through astrocyte–neuron lactate shuttle (ANLS) [119,120]. C10 may also restore synaptic function by inhibiting mTOR signaling and regulating the expression of AMPA receptor subunit (e.g., GluA2) [121]. Flow data suggest that higher plasma levels of C8 and DHA are independently associated with a lower risk of MCI [122]. However, this protective effect may be limited to the pre-disease stage, with little effect on the progression from diagnosed MCI to AD [123].

### 4.2. PD and Amyotrophic Lateral Sclerosis (ALS)

PD often manifests with non-motor symptoms in its early stages, including reduced sense of smell, fatigue, depression and behavior changes. Motor symptoms include slowness of movement, muscle stiffness, resting tremors, and instability and difficulty swallowing in late stages [124]. The disease is characterized by progressive loss of dopaminergic neurons, resulting in impaired striatal projection to the putamen, and is often associated with dementia and depression in late stages (Figure 2A) [125]. Recent research suggests that PD pathology may originate in the enteric nervous system and spread to the central nervous system. C8 can reduce lipid peroxidation and inflammatory markers in brain–gut axis tissue exposed to neurotoxins [126]. Acute C8 intake may also relieve dopamine neurotransmitter damage and enhance mitochondrial metabolism in the striatum (Table 2) [75]. C10 also plays a neuroprotective role in PD models by inhibiting insulin/IGF-1 signaling, activating transcription factor DAF-16, and upregulating the expression of antioxidant and molecular chaperone genes [76]. An analysis of 117 PD patients indicated that moderate intake of C8 and C10 (averaging 0.2 and 0.4 g/day, respectively) might be beneficial, while excessive intake could impair motor function (Table 1) [28].

ALS is a fatal motor neuron disease characterized by progressive degeneration of motor neurons in the brain and spinal cord (Figure 2C), leading to loss of nerve control, weakness, paralysis, respiratory failure and eventually death [127]. MCTs have the potential to improve motor function through mechanisms that may involve maintaining neuromuscular junction integrity, regulating synaptic plasticity, and modulating glutamate transmission [128]. In symptomatic SOD1-G93A transgenic ALS mice, caprylic triglyceride alleviated motor deficits by enhancing mitochondrial oxygen consumption and restoring energy metabolism (Table 2) [77]. In a fruit fly model of TDP-43 proteinopathy, MCTs provided an alternative fuel, alleviating mitochondrial energy deficits and thereby improving motor neuron function and neuromuscular junction integrity [78]. In the C9-ALS/FTD fruit fly model, two MCFAs (nonanoic acid and 4-methyloctanoic acid (4-MOA)) exerted neuroprotective effects through presynaptic and postsynaptic mechanisms, respectively, jointly alleviating motor dysfunction, neuromuscular junction degeneration, and metabolic disorders [79]. These findings position MCTs as a multi-target strategy for ALS, modulating neuronal excitability and energy homeostasis.

### 4.3. Epilepsy

Epilepsy, one of the most common disorders of the nervous system worldwide, is characterized by recurrent episodes of abnormal, synchronous electrical activity in the brain (Figure 2D). During interictal periods, persistent changes in brain function may increase the risk of psychiatric and neuropsychiatric disorders, especially in patients with drug-resistant epilepsy (DRE). Epilepsy management requires not only seizure control but also attention to comorbid mental health issues, with depression, anxiety, and autism being the most common neuropsychiatric comorbidities [129]. MCTs demonstrate multifaceted potential in the treatment of epilepsy, involving mechanisms such as metabolic regulation, neuroprotection, and behavioral improvement [130].

Recent studies have highlighted the special effects of C10. It can directly antagonize AMPA receptors, inhibiting spontaneous epileptiform activity and excitatory synaptic transmission in human DRE cortical tissue, a mechanism shared with the antiepileptic drug perampanel [131]. C10 also upregulates key autophagy proteins (Atg1, Atg8), specifically inducing autophagic flux, and its effect is comparable to that of other anticonvulsant compounds [132]. Of note, clinical efficacy is not always associated with elevated serum C10 levels, suggesting that its effects may involve indirect or central mechanisms beyond peripheral concentrations [133]. The effects of C10 are dose-dependent: long-term dosing (3.0 mmol/kg for 21 days) reduced body weight and improved depressive-like behavior in rodents, while acute high dose (30 mmol/kg) reduced weight, inhibited locomotor activity, and increased anxiety-like behavior [80].

Animal models further elucidate the possible mechanisms by which MCTs improve epilepsy and its comorbidity. In canine spontaneous epilepsy, MCTs induce ketosis without severe carbohydrate restriction [134]. In pentylenetetrazol-induced seizures in mice, MCTs reduced seizure severity and comorbid depressive-like behaviors by inhibiting mTOR and GSK-3β signaling and reducing hippocampal lipid peroxidation (Table 2) [81]. In addition, an exogenous ketone supplement (KEMCT) improved anxiety-like behaviors in rats [135].

Clinically, approximately 65% of patients tolerated MCT supplementation well [136]. As listed in Table 1, MCTs can significantly reduce seizure frequency, and some patients even achieve seizure freedom, mainly by improving brain energy metabolism and gut–brain axis function [29]. This is associated with increased levels of ketone bodies, fatty acid complexes, branched-chain amino acid catabolites, and GABAergic metabolites [30]. One notable case report described a child with comorbid autism and epilepsy who achieved seizure freedom and significant autism symptom improvement on a gluten-free/casein-free diet supplemented with MCTs, suggesting that MCTs may have broader benefits for neurodevelopmental disorders [31].

The evidence for MCTs in AD and PD remains largely preclinical (in vitro and animal models) or derived from small-scale clinical studies with limited sample sizes, and large-scale randomized controlled trials are lacking. For epilepsy, the evidence is relatively stronger, with several clinical studies (mostly open-label or retrospective) and the established use of ketogenic diets. However, randomized controlled trials specifically evaluating MCTs (rather than classic ketogenic diets) are still limited. Regarding the case report on autism, although relevant validation exists, further verification in controlled studies is needed.

## 5. Effects of MCTs on Gut Health

The beneficial effects of MCTs, MCFAs, and their derivatives, such as medium-chain monoglycerides (MCMs, e.g., glyceryl monocaprylate, glyceryl monocaprate, monolaurin), on gut health extend beyond their unique direct absorption pathway. They play a key role in the management of digestive diseases and maintenance of systemic homeostasis by regulating intestinal microbiota and barrier function, improving intestinal dysbiosis, and repairing intestinal barriers.

### 5.1. MCTs in the Management of Digestive and Malabsorptive Disorders

MCTs have important value in nutritional interventions for a variety of digestive and absorptive disorders. They provide a core energy solution for diseases related to chylomicron transport disorders. As shown in Table 1, in the treatment of postoperative chylous leaks and chylothorax, a diet rich in MCTs can effectively reduce lymphatic fluid production, maintain nutritional and fluid balance, and, when combined with enteral nutrition support, avoid secondary surgery in most patients, representing an effective non-surgical treatment strategy [32,33,34]. For inherited diseases such as familial chylomicronemia syndrome, long-term restriction of long-chain fats and supplementation of MCTs is a cornerstone therapy for preventing acute pancreatitis triggered by inherited hypertriglyceridemia [35,36]. When intestinal function itself is impaired, MCTs directly support intestinal epithelial repair and nutrient absorption. In patients with environmental enteropathy, short bowel syndrome, or severe pancreatic insufficiency, MCTs bypass the barrier of long-chain fat digestion and absorption, are taken up directly by intestinal epithelial cells, and are transported via the portal vein, providing highly efficient energy without increasing intestinal burden [37]. MCTs can upregulate the expression of key intestinal nutrient transporters (e.g., SLC6A4, SLC7A9), directly promoting the recovery of absorptive function in patients with intestinal failure [137].

### 5.2. Regulatory Effects of MCT Metabolites on Gut Health

#### 5.2.1. MCFAs: Modulating Gut Microbiota and Barrier Function

MCFAs have inhibitory and regulatory effects on gut microbes, showing activity against a variety of bacteria (including *Escherichia coli*, *Staphylococcus aureus*, and *Salmonella biofilms*), fungi (such as *Candida albicans*), and even enveloped viruses [138,139,140,141]. This antibacterial effect helps reduce pathogen biofilm formation and proliferation. In recent years, researchers have engineered probiotic yeast strains that stably produce and secrete MCFAs through metabolic engineering, advancing the development of engineered prophylactic intervention [142]. MCFAs can also optimize microbial communities, reduce the abundance of potentially harmful bacteria (e.g., *Bacteroides* spp., *Vibrionaceae*) and increase beneficial species (e.g., *Blautia*, *Fusicatenibacter*, and *actinomycetes*), thereby enhancing gut barrier function, host immunity, and overall gut health (Table 2) [82,83].

#### 5.2.2. MCMs: Modulating Gut Microbiota and Barrier Function

Derivatives of MCFAs, specifically MCMs, have direct and potent roles in regulating microbiota and repairing barriers. In terms of microbiota regulation, MCMs can selectively reshape gut microorganisms, inhibit the proliferation of potential pathogens, and significantly promote the growth of beneficial bacteria such as *Bacillus*, *Rhodobacteraceae*, and *Lactobacillus* [83,143]. This shift enhances the capacity to produce short-chain fatty acids (SCFAs), optimizes the gut metabolic environment for amino acids and carbohydrates, and maintains ecological balance and stability [144,145].

In the enhancement and repair of the gut barrier, different MCMs act through multiple pathways. Octanoate can effectively repair gut damage and strengthen the physical barrier by upregulating the expression of tight junction protein zonula occludens-1 (ZO-1) expression, reducing reactive oxygen species (ROS) levels, and inhibiting pro-inflammatory factors [146]. Glycerol monolaurate directly repairs barrier structure by upregulating the expression of Mucin 2 and Claudin-1, and exerts strong anti-inflammatory and antioxidant effects by modulating signaling pathways such as toll-like receptor 4/nod-like receptor family pyrin domain-containing 3 (TLR4/NLRP3) and AMPKα1, thereby improving intestinal follicle morphology and overall health [147]. Additionally, derivatives such as sodium caprate have also been shown to promote gut tissue development, enhance barrier function, and increase antioxidant capacity [148].

The evidence for MCTs/MCFAs/MCMs in gut health is supported by a combination of in vitro antimicrobial assays, animal studies, and clinical applications (e.g., in chylous leakage, short bowel syndrome). The engineered probiotic approach is at an early preclinical stage. Human randomized controlled trials specifically evaluating gut microbiota modulation by MCTs are limited but growing.

## 6. Potential Effects of MCTs on Improving Muscle Mass and Function

MCTs have been studied for their roles in regulating muscle metabolism, combating sarcopenia, and enhancing athletic performance. Increasing attention has been paid to different-chain-length MCFAs (such as C8, C10, C12) that regulate mitochondrial function and protein homeostasis through multi-target mechanisms, showing great potential for aging, disease, and exercise interventions.

### 6.1. Cell and Animal Model Mechanisms

In skeletal and cardiac muscle cells, C8 is more effective than C10 and C12 in enhancing mitochondrial biosynthesis and ameliorating myocardial injury. In skeletal muscle cells, C8 significantly enhances mitochondrial autophagy (via the PTEN/Parkin pathway), promotes mitochondrial biogenesis (upregulating PPARγ and PGC-1α), and upregulates muscle differentiation markers (e.g., myogenic differentiation 1 (MyoD)) and oxidative phosphorylation, thereby resisting muscle atrophy [149]. In cardiomyocytes, C8 inhibits high-mobility group box 1 (HMGB1), alleviates mitochondrial oxidative stress, maintains membrane potential, and improves myocardial injury [150]. In rat aortic smooth muscle cells (RASMC) induced by tunicamycin, both C8 and C10 relieved endoplasmic reticulum stress, reduced oxidative damage, inhibited apoptosis, and effectively protected vascular smooth muscle cells from pathological damage [151].

In animal models, MCT supplementation shows clear advantages for muscle function. In obese rat models, 12-week MCT intervention significantly improved grip strength and exercise endurance, possibly due to inhibition of the ubiquitin–proteasome pathway (UPP) and promotion of muscle regeneration. However, excessive intake (e.g., 40% of diet) weakened these benefits [152]. In aged BRD4-deficient mice, long-term MCT intake prevents fat accumulation and skeletal muscle atrophy by enhancing energy metabolism and regeneration in fast-twitch (gastrocnemius) muscle and suppressing degradation in slow-twitch (soleus) muscle (Table 2) [84]. In sedentary mice, MCTs activated the AMPK pathway, reshaped muscle fatty acid composition, enhanced liver gluconeogenesis, and improved energy metabolism and exercise endurance [85]. Some studies suggest that while C8 and C10 reduce weight and increase ketone levels, they may not reverse and may even exacerbate age-related bone loss [153].

### 6.2. Clinical Applications and Intervention

The molecular mechanisms revealed in cell and animal models have been partially supported by clinical studies. Below, we discuss the applicability of MCTs in different populations, focusing on dosage and combined interventions (Figure 1).

Three randomized controlled trials (RCTs) in frail older adults aged 85 and over found that supplementation with 6 g of MCT for 3 months significantly improved muscle function, increased right arm muscle area, enhanced grip strength and lower limb function, and reduced triceps skinfold thickness [38]. When combined with moderate-intensity exercise (e.g., walking), MCTs enhance knee extension strength in middle-aged and elderly individuals with low BMI, effectively preventing muscle strength loss [39]. A number of studies suggest that MCTs may activate the sympathetic nervous system, increase plasma-acylated ghrelin concentrations, and utilize the nitrogen-sparing effect of ketones to promote mitochondrial biosynthesis and maintain muscle protein balance, both of which are key to improving muscle mass and function [154].

For stroke patients, an intervention combining 40 g/day MCT with high-frequency chair–standing training significantly improved motor scores, grip strength, and skeletal muscle index on the Functional Independence Measure. This regimen reversed post-stroke muscle loss and dysfunction, markedly enhancing rehabilitation [40]. For patients with rheumatoid arthritis (RA), supplementation with 30 g/day MCTs for 16 weeks significantly reduced disease activity (assessed by SDAI score) and increased ketone levels; the effect was even more pronounced when combined with 30 g of dietary fiber [41]. For specific metabolic disorders such as LCFA oxidation disorders, odd-carbon MCTs (e.g., triheptanoin) can be used as alternative energy substrates, effectively reducing acute metabolic crises, improving or even reversing cardiomyopathy and arrhythmia in pediatric patients, and demonstrating good long-term safety. For premature infants at metabolic risk, supplementation with MCT oil or low-fat MCT-fortified formulas can help maintain growth and improve metabolic indicators; however, the efficacy in severely ill newborns still needs further validation [42,43,44,45].

Cellular and animal studies provide relatively robust mechanistic evidence for MCTs in muscle metabolism, though most are acute or short-term (weeks to months). Long-term safety data (e.g., on bone health) remain conflicting. The clinical evidence for MCTs in muscle function is promising, particularly in frail older adults and post-stroke rehabilitation, supported by several RCTs (though mostly small to medium sized, *n* < 100). Evidence in rheumatoid arthritis and metabolic disorders is more preliminary. Larger, longer-term (>6 months) multi-center trials are needed.

## 7. Conclusions and Perspectives

### 7.1. Conclusions

This review systematically elaborates on the important roles of MCTs and their bioactive metabolites in various physiological and pathological processes. Due to their rapid energy supply, efficient metabolism, and multi-target regulatory properties, MCTs demonstrate unique application value in metabolic health, neuroprotection, gut homeostasis, and muscle function maintenance. In metabolic diseases, MCTs provide nutritional regimens for obesity, T2DM, and metabolic liver diseases by stimulating thermogenesis in adipose tissue, enhancing insulin signaling, increasing energy expenditure, and reducing liver lipid accumulation and inflammation. In neurodegenerative diseases, MCTs provide efficient alternative energy sources to the brain through ketogenic effects, and also exert synergistic neuroprotective effects, including anti-inflammatory, antioxidant, autophagy regulation, and synaptic protection, bringing new hope for the adjunctive treatment of AD, PD, and drug-resistant epilepsy. In gut health, MCTs and their derivatives (e.g., MCMs) alleviate malabsorption disorders, enhance intestinal barrier integrity through direct antimicrobial activity and microbiome modulation, and play a protective role in a variety of digestive diseases. In muscle health, MCTs provide a nutritional rationale for counteracting age- or disease-related sarcopenia. They improve muscle strength and exercise endurance by optimizing mitochondrial function via pathways such as AMPK/PGC-1α, and regulate the balance between protein synthesis and degradation.

### 7.2. Perspectives

Despite the increasing number of available studies, research in this area still faces several limitations and challenges. First, available studies suggest that high doses of MCTs may cause gastrointestinal discomfort (e.g., nausea, diarrhea); long-term use may adversely affect bone health (e.g., exacerbate age-related bone loss). Furthermore, individual responses to MCTs vary significantly. For example, APOE ε4 carriers show less improvement in cognitive outcomes, sex and baseline body weight influence energy expenditure, and gut microbiota composition may further modulate the metabolic effects of MCTs. These issues require further experimental investigation. Second, there are constraints on the scale and duration of clinical evidence. Most clinical studies are limited by small sample sizes and short intervention periods, and lack large-scale, long-term, randomized controlled trial data. This limits the comprehensive evaluation of the long-term efficacy and safety of MCTs. Third, application regimens are insufficiently precise. For different populations (e.g., varying ages, genotypes, disease states, and severity), the optimal dosage of MCTs, fatty acid chain-length ratios (e.g., C8:C10), and intervention duration remain unclear. Evidence-based, personalized application guidelines are lacking. Fourth, exploration of synergistic effects is limited. Research on the synergistic effects between MCTs and other dietary components (e.g., dietary fiber, ω-3 polyunsaturated fatty acids, polyphenols) or non-pharmacological interventions (e.g., exercise) is at an early stage, hindering the development of more effective integrated intervention strategies.

To address these limitations and promote the development of the field, future research should focus on the following directions. First, using multi-omics approaches (e.g., metabolomics, proteomics, and systems biology), we can further elucidate the complex metabolic networks and molecular mechanisms of MCTs in vivo and find specific biomarkers that predict the effects of their interventions, thereby accelerating the translation of basic research results into clinical applications. Second, we should conduct large-scale, long-term, and rigorously designed randomized controlled trials to determine the effective dosage, long-range safety and health benefits of MCTs in the prevention and treatment of various chronic diseases. Third, precision nutrition research combining genomics and metabolomics technologies should be advanced to explore the impact of individual differences (e.g., APOE genotype, gut microbiota composition) on the interventional effects of MCTs, providing a basis for developing personalized nutrition strategies. Fourth, the combined effects of MCTs with other dietary components or lifestyle interventions should be systematically evaluated to develop multimodal integrated health management programs based on MCTs. In summary, MCTs have broad prospects for chronic disease prevention and adjunctive therapy. Through interdisciplinary collaboration and systematic resolution of the key scientific issues outlined above, MCTs are expected to play an increasingly important role in the fields of precision nutrition and metabolic medicine.

## Figures and Tables

**Table 1:** Clinical applications of MCTs in different populations.

Population Group	MCT Dosage/Intervention	Reported Effects	References
Healthy individuals	30 g/day, 4 weeks	No adverse effects; good physiological tolerance	[16]
Sedentary obese individuals	2 weeks	Increased postprandial energy expenditure	[17]
Obese patients (female)	4 weeks	Higher energy expenditure in those with higher body weight	[18]
Obese patients (male)	4 weeks	Higher energy expenditure in those with lower body weight	[19]
Patients with non-insulin-dependent diabetes mellitus	4 weeks	Attenuated postprandial blood glucose fluctuations	[20]
CPT1A deficiency	——	Bypasses defective metabolic process	[21]
Neonatal intrahepatic cholestasis (citrin deficiency)	——	Improved cholestasis and coagulation function	[22]
Children with biliary atresia	——	Supports preoperative nutrition	[23,24]
Patients with mild cognitive impairment (MCI)	30–42 g/day, 6–9 months	Stable or improved cognitive function	[25,26]
Patients with mild to moderate AD/MCI	20–70 g/day	Stable or improved cognitive function	[27]
Patients with Parkinson’s disease (PD)	0.2–0.4 g/day	Improved motor function	[28]
Patients with drug-resistant epilepsy	——	Reduced seizure frequency, some achieved seizure freedom	[29,30]
Child with comorbid autism and epilepsy	——	Seizure freedom, improved autism symptoms	[31]
Patients with postoperative chylous leakage/chylothorax	——	Maintained nutritional and fluid balance	[32,33,34]
Familial chylomicronemia syndrome	——	Prevents acute pancreatitis, supports nutrient absorption	[35,36]
Environmental enteropathy/short bowel syndrome/severe pancreatic insufficiency	——	Direct uptake by intestinal epithelial cells, providing efficient energy	[37]
Frail older adults aged ≥85 years	6 g/day, 3 months	Significantly improved muscle function	[38]
Middle-aged and elderly with low BMI	——	Improved knee extension strength	[39]
Stroke patients	40 g/day	Reversed post-stroke muscle loss and dysfunction	[40]
Patients with rheumatoid arthritis (RA)	30 g/day, 16 weeks	Reduced disease activity, increased ketone levels	[41]
Long-chain fatty acid (LCFA) oxidation disorders/premature infants at metabolic risk	——	Reduced acute metabolic crises/maintained growth and improved metabolic indicators	[42,43,44,45]

**Table 2:** Mechanisms of positive/negative effects of MCTs on chronic diseases.

Health Domain	Key Findings	Level of Evidence/Study Type	Limitations	References
Obesity	MCTs reduce body weight by increasing energy expenditure, suppressing appetite, promoting fat breakdown, and inhibiting fat synthesis	Animal studies + small-scale clinical studies (*n* < 50, 2–4 weeks)	Small sample size, short intervention duration; effects influenced by sex and baseline body weight	[65,66,67,68]
T2DM/insulin resistance	Improve insulin sensitivity, attenuate postprandial blood glucose fluctuations	Animal studies + in vitro studies + small-scale clinical studies (*n* < 50, 4 weeks)	Small sample size, short intervention duration; C12 may induce insulin resistance; lack of long-term glycemic evidence	[69,70]
CPT1A deficiency, etc.	Provide nutrition as an alternative energy substrate	Multiple clinical reports confirmed	Specific to inherited metabolic liver diseases	[21]
MASLD/MASH	Reduce hepatic lipid accumulation, improve local liver inflammatory microenvironment	Animal studies + in vitro studies	Insufficient clinical study results	[71,72]
AD/MCI	Improve cognitive function, enhance brain ketone utilization	Small-scale clinical studies (20–70 g/day, up to 9 months) + meta-analysis + animal studies	Lack of large-scale clinical validation; cognitive improvement may be limited to specific domains and disease stages	[73,74]
PD	Reduce dopaminergic neuron damage, improve motor function	Animal studies + small-sample clinical study (117 PD patients)	Excessive intake of C8/C10 may impair motor function	[28,75,76]
ALS	Improve motor neuron function, maintain neuromuscular junction integrity	Animal studies (SOD1-G93A mice, TDP-43/C9 Drosophila models)	No human clinical studies	[77,78,79]
Epilepsy	Reduce seizure frequency, some patients become seizure-free	Animal studies + clinical studies + case reports	Uncertain MCT dosage for dietary intervention	[80,81]
Gut health	Improve malabsorption, repair gut barrier, modulate gut microbiota	In vitro studies + animal studies + clinical applications	Application is relatively disease-specific; engineered probiotics are at a pre-clinical stage	[82,83]
Muscle health	Improve muscle strength, function, and exercise endurance	In vitro studies + animal studies + small to medium clinical studies	Long-term safety needs further validation	[84,85]

## Data Availability

No new data were created or analyzed in this study. Data sharing is not applicable to this article.
